# Homozygosity Mapping Reveals Population History and Trait Architecture in Self-Incompatible Pear (*Pyrus* spp.)

**DOI:** 10.3389/fpls.2020.590846

**Published:** 2021-01-05

**Authors:** Satish Kumar, Cecilia Hong Deng, Martin Hunt, Chris Kirk, Claudia Wiedow, Daryl Rowan, Jun Wu, Lester Brewer

**Affiliations:** ^1^Hawke’s Bay Research Centre, The New Zealand Institute for Plant and Food Research Limited, Havelock North, New Zealand; ^2^Mount Albert Research Centre, The New Zealand Institute for Plant and Food Research Limited, Auckland, New Zealand; ^3^Palmerston North Research Centre, The New Zealand Institute for Plant and Food Research Limited, Palmerston North, New Zealand; ^4^Centre of Pear Engineering Technology Research, Nanjing Agricultural University, Nanjing, China; ^5^Motueka Research Centre, The New Zealand Institute for Plant and Food Research Limited, Motueka, New Zealand

**Keywords:** *Pyrus*, runs of homozygosity, signatures of selection, genotyping-by-sequencing, inbreeding, germplasm, quantitative trait loci, genome wide association study

## Abstract

Runs of homozygosity (ROH) have been widely used to study population history and trait architecture in humans and livestock species, but their application in self-incompatible plants has not been reported. The distributions of ROH in 199 accessions representing Asian pears (45), European pears (109), and interspecific hybrids (45) were investigated using genotyping-by-sequencing in this study. Fruit phenotypes including fruit weight, firmness, Brix, titratable acidity, and flavor volatiles were measured for genotype-phenotype analyses. The average number of ROH and the average total genomic length of ROH were 6 and 11 Mb, respectively, in Asian accessions, and 13 and 30 Mb, respectively, in European accessions. Significant associations between genomic inbreeding coefficients (F_ROH_) and phenotypes were observed for 23 out of 32 traits analyzed. An overlap between ROH islands and significant markers from genome-wide association analyses was observed. Previously published quantitative trait loci for fruit traits and disease resistances also overlapped with some of the ROH islands. A prominent ROH island at the bottom of linkage group 17 overlapped with a recombination-supressed genomic region harboring the self-incompatibility locus. The observed ROH patterns suggested that systematic breeding of European pears would have started earlier than of Asian pears. Our research suggest that F_ROH_ would serve as a novel tool for managing inbreeding in gene-banks of self-incompatible plant species. ROH mapping provides a complementary strategy to unravel the genetic architecture of complex traits, and to evaluate differential selection in outbred plants. This seminal work would provide foundation for the ROH research in self-incompatible plants.

## Introduction

Systematic genetic improvement of outbred plants for economically important traits such as yield, consumer acceptance and nutritional value, has led to the loss of genetic diversity within and among accessions in domesticated gene pools (Van de Wouw et al., 2010; Smýkal et al., 2018). Mating among related individuals would lead to inbreeding, which increases the level of homozygosity and reduces recombination frequency in the genome (Charlesworth, 2003). In addition to inbreeding, long tracts of consecutive homozygous segments in the genome can arise through mechanisms such as natural and artificial selection, genetic drift and population bottlenecks (Ceballos et al., 2018). Runs of homozygosity (ROH), first described by Gibson et al. (2006), are successive homozygous segments of the genome where the two haplotypes inherited from the parents are identical-by-descent. Consanguinity would result in long ROH, whereas larger populations have fewer, shorter ROH (Ceballos et al., 2018).

As recombination interrupts long chromosome segments over time, the length of ROH segment depends in part on the number of generations since the parents shared an ancestor in common (Curik et al., 2014). In an inbred population we would expect to see longer homozygous segments than in outbred populations. Long ROH could still be observed in outbred accessions, perhaps due to unusual mutation and recombination suppression at certain genomic locations. The shorter ROH would indicate the presence of more ancient relatedness which is unaccounted for in the absence of the individual’s historic pedigree record. Therefore, the extent and frequency of ROH could reveal population history of a species, such as inbreeding, change of population size, and admixture (Ceballos et al., 2018; Clark et al., 2019). ROH mapping also allows a comparison of the degree of homozygosity among populations with varying degrees of isolation and inbreeding (Kirin et al., 2010).

With the development of cost-effective genome sequencing technologies, large numbers of single nucleotide polymorphisms (SNPs) can be generated at a relatively low price. This facilitates ROH analysis to capture the genomic regions contributing to inbreeding, and thus to assess the breeding history and to identify the genetic components for trait selection. ROH were first recorded in humans by using 8,000 short tandem-repeat polymorphisms (Broman and Weber, 1999). Using ∼700,000 SNPs, Gibson et al. (2006) reported the widespread occurrence of ROH in humans and revealed the harmful effects of recessive deleterious variants present in the ROH regions. Clark et al. (2019) showed genomic inbreeding coefficients (F_ROH_) derived from ROH, were significantly associated with deleterious effects in humans.

The ROH patterns were shown to differ markedly among cattle breeds (Purfield et al., 2012), and the genomic regions with significant excesses of ROH (termed as ROH islands; Curik et al., 2014) were reported to be associated with signatures of positive selection in horses (Metzger et al., 2015; Grilz-Seger et al., 2019). Beynon et al. (2015) used ROH to reveal population history and structure in a sheep population. ROH analysis was shown to be in agreement with other approaches (e.g., genome-wide association (GWA); haplotype analysis and signatures of selection) to identify the SLICK hair locus in cattle (Huson et al., 2014). Biscarini et al. (2014) also showed agreement between ROH-based and GWA methods to identify quantitative trait loci (QTLs) in farm animals. ROH-guided analyses have also been shown to be a reliable tool for the design of mating schemes to minimize inbreeding (Toro and Varona, 2010; Biscarini et al., 2014).

Inbreeding usually results in the loss of vigor and reduced reproductive fitness of offspring in outbred plant species (Moore and Janick, 1975; Angeloni et al., 2011). Lander and Botstein (1987) suggested that the deleterious recessive variants can be identified in inbred individuals by the presence of long homozygous regions. In the process of evolution and the development of new cultivars under the influence of different mating systems, directional selection, different population sizes and development histories would generate unique ROH distribution patterns in the plant genome; therefore, the number, length, distribution and frequency of ROH in plant genomes would provide rich genetic background information, such as population histories and inbreeding levels.

Despite many studies in humans and livestock populations, the use of ROH to infer inbreeding, population history and trait architecture has apparently not been explored in outbred plant species. The availability of reference genomes and cost-effective genotyping technologies provide an excellent opportunity to evaluate the use of ROH, which still appears an unexplored research field in outbred plants. Pear (*Pyrus* spp.), which exhibits gametophytic self-incompatibility, is among the important temperate fruit tree species, with at least 3,000 years of cultivation history. The genus *Pyrus* is believed to have originated in the mountainous regions of western China (Wu et al., 2018). Pear is commercially grown in more than 50 countries in different geographical regions, but *Pyrus communis* is the predominant species cultivated in Europe, and the major cultivated species in Asia include *P*. *pyrifolia*, *P*. bretschneideri, *P. sinkiangensis*, and *P*. *ussuriensis*. Asian pears display a crisp texture, while the European pear is well known for buttery and juicy texture. Various pear breeding programs use interspecific hybrids to develop cultivars with novel combinations of texture and flavor (Brewer and Palmer, 2010).

Natural and artificial selection, as well as independent evolution, has resulted in *Pyrus* species that differ extensively especially in their fruit characteristics (Wu et al., 2018). Different pear species could conceptually be considered as subpopulations, so investigation of ROH patterns would provide insight into their disparate histories. Here we use *Pyrus* as an example to demonstrate application of the ROH concept to investigate population history and trait architecture in self-incompatible outbred plant species.

## Materials and Methods

### Plant Material, Phenotyping, and Genotyping

Accessions of the European and Asian pear species were imported into New Zealand to initiate an interspecific hybrid breeding program in 1983 (Brewer and Palmer, 2010). The successive generations of hybrids were mainly developed from crosses among a few selected hybrids from the previous generation. The imported accessions, as well as the selections from the interspecific hybrid program were propagated over a number of years and planted in duplicate at the Plant and Food Research (PFR) Pear Repository for further assessment and long-term conservation. All trees received standard orchard management for nutrition, pesticide, and irrigation. Six fruit from each plant in the repository were harvested over two consecutive years 2014 and 2015. An average value of six fruits was used to represent each phenotype of each accession.

For the purpose of this study, a total of 199 accessions, including 46 representing Asian species (36 *P. pyrifolia*, 10 *P. × bretschneideri*), 108 of European pear (*P*. *communis*), and 45 hybrids between Asian and European species (Table 1) were sampled. Protocol for fruit harvesting and assessment were as reported earlier (Kumar et al., 2017); six fruit from each accessions were stored for 28 days at 3°C, then a further 1 day at 20°C before evaluation. Skin russet coverage (RUS), sensory flavor intensity (FINT) and skin bitterness (BIT) were scored on intensity scales where 0 = none and 9 = highest. Scuffing (SCUF) was also rated on a 0–9 scale (0 = no darkening; 9 = solid brown or black coloration) after each fruit was firmly rubbed across the cup of a molded pulp fibreboard fruit packing tray and assessed after 2 h. Fruit shape index (SHAP) was measured using a two dimensional shape chart and fruit weight (AVFW) was recorded as the average weight of the six fruit. Fruit firmness (FF) was determined on opposite sides of each fruit after peel removal using a Fruit Texture Analyzer (GÜSS) fitted with an 11 mm diameter probe tip. Soluble solids concentration (SSC) for each fruit was measured, with the juice expressed during the firmness probe, using a digital refractometer (Atago PR-32). Bulked juice from the cortical flesh of the sample fruit was used to measure titratable acidity (TA) using an automatic acid titrator (Metrohm 716 DMS) and the percentage of malic acid in fruit juice was recorded.

**TABLE 1:** List of pear accessions and their *Pyrus* species group.

**CULTIVAR/Selection**	**Species**	**CULTIVAR/Selection**	**Species**
3189	*P. communis*	NOUVEAU POITER	*P. communis*
2-301	*P. communis*	OLD HOME	*P. communis*
6/23/94	*P. communis*	OTTAWA-291	*P. communis*
6-31-100	*P. communis*	OVID	*P. communis*
6-31-68	*P. communis*	C01	*P. communis*
ANGELYS	*P. communis*	C02	*P. communis*
Aurora	*P. communis*	C03	*P. communis*
AUTUMN BERGAM	*P. communis*	C04	*P. communis*
BEURRE BOSC	*P. communis*	C05	*P. communis*
BEURRE CAPIAMONT	*P. communis*	C06	*P. communis*
BEURRE EASTER	*P. communis*	C07	*P. communis*
BEURRE HARDY	*P. communis*	C08	*P. communis*
BROCKWORTH	*P. communis*	C09	*P. communis*
BROWN BEURRE	*P. communis*	C10	*P. communis*
BUTIRRA PRECOCE MORRETINI	*P. communis*	C11	*P. communis*
BUTIRRA ROSATA MORRETINI	*P. communis*	C12	*P. communis*
CALIFORNIA	*P. communis*	C13	*P. communis*
CARMEN	*P. communis*	C14	*P. communis*
COLETTE	*P. communis*	C15	*P. communis*
CONCORDE	*P. communis*	C16	*P. communis*
CRIMSON GEM COMICE	*P. communis*	C19	*P. communis*
D’Incontinue	*P. communis*	C20	*P. communis*
DOYENNE DU COMICE	*P. communis*	C24	*P. communis*
ELDORADO	*P. communis*	C25	*P. communis*
Elizabeth Cole	*P. communis*	C26	*P. communis*
FERTILITY	*P. communis*	C27	*P. communis*
FLEMISH BEAUTY	*P. communis*	P327-57	*P. communis*
FLORIDA HOME	*P. communis*	PACKHAM’S TRIUMPH	*P. communis*
Gleau Morceau	*P. communis*	PASSA CRASSANA	*P. communis*
GOLDEN RUSSET BOSC	*P. communis*	PATRICK BARRY	*P. communis*
GORHAM	*P. communis*	PATTEN	*P. communis*
GRAND CHAMPION	*P. communis*	Peamy	*P. communis*
HARROW DELIGHT	*P. communis*	PIERRE CORNEILLE	*P. communis*
HIGHLAND	*P. communis*	PRESIDENT D’OSMOND	*P. communis*
HOSKINGS	*P. communis*	President Heron	*P. communis*
HOWELL	*P. communis*	PRINCESS	*P. communis*
HW606 (Harovin Sundown)	*P. communis*	PT AV-63-2076	*P. communis*
JUMBO (STARKS)	*P. communis*	RED ANJOU	*P. communis*
Jupp	*P. communis*	RED SENSATION BARTLETT	*P. communis*
LOUISE BON DE JERSEY	*P. communis*	REIMER RED	*P. communis*
Margeurite Marrilat	*P. communis*	ROGUE RED	*P. communis*
MAX RED BARTLETT	*P. communis*	ROSEMARIE	*P. communis*
Moders	*P. communis*	Ruby	*P. communis*
MOONGLOW	*P. communis*	RX359	*P. communis*
Nellie	*P. communis*	RX529	*P. communis*
New York	*P. communis*	RX810	*P. communis*
SIERRA	*P. communis*	Shingo	*P. pyrifolia*
SILVERBELL	*P. communis*	SHINKO	*P. pyrifolia*
STARKING DELICIOUS	*P. communis*	SHINSUI	*P. pyrifolia*
STARKRIMSON	*P. communis*	SUISEI	*P. pyrifolia*
SUPER COMICE	*P. communis*	TAMA	*P. pyrifolia*
SWISS BARTLETT	*P. communis*	WASEAKA	*P. pyrifolia*
TAYLORS GOLD	*P. communis*	Yasato	*P. pyrifolia*
TENN	*P. communis*	B01	*P. bretschneideri*
TN09-46	*P. communis*	B02	*P. bretschneideri*
TOSCA	*P. communis*	B03	*P. bretschneideri*
US307	*P. communis*	PINGGUOLI	*P. bretschneideri*
US56112/46	*P. communis*	QIYUESU	*P. bretschneideri*
UVEDALES ST GERMAINE	*P. communis*	TSULI	*P. bretschneideri*
Velvetine	*P. communis*	XINYALI	*P. bretschneideri*
WINTER NELIS	*P. communis*	XUEHUALI	*P. bretschneideri*
WORDEN SECKLE	*P. communis*	YALI	*P. bretschneideri*
Cangxili	*P. pyrifolia*	Crispie	Hybrid
Choju	*P. pyrifolia*	HWA HONG	Hybrid
CHOJURO	*P. pyrifolia*	MAXIE	Hybrid
DAN BAE	*P. pyrifolia*	H01	Hybrid
DOITSU	*P. pyrifolia*	H02	Hybrid
GION	*P. pyrifolia*	H03	Hybrid
Gold Nijisseiki	*P. pyrifolia*	H04	Hybrid
HAKKO	*P. pyrifolia*	H05	Hybrid
HEISHI	*P. pyrifolia*	H06	Hybrid
HOKUSEI	*P. pyrifolia*	H07	Hybrid
Hougetsu	*P. pyrifolia*	H08	Hybrid
IMAMURA AKI	*P. pyrifolia*	H09	Hybrid
NIITAKA	*P. pyrifolia*	H10	Hybrid
P01	*P. pyrifolia*	H11	Hybrid
P02	*P. pyrifolia*	H12	Hybrid
P03	*P. pyrifolia*	H13	Hybrid
P04	*P. pyrifolia*	H14	Hybrid
P05	*P. pyrifolia*	H15	Hybrid
P07	*P. pyrifolia*	H16	Hybrid
P08	*P. pyrifolia*	H17	Hybrid
P09	*P. pyrifolia*	H18	Hybrid
P10	*P. pyrifolia*	H19	Hybrid
P11	*P. pyrifolia*	H20	Hybrid
P12	*P. pyrifolia*	H21	Hybrid
P13	*P. pyrifolia*	H22	Hybrid
P14	*P. pyrifolia*	H23	Hybrid
P15	*P. pyrifolia*	H27	Hybrid
P16	*P. pyrifolia*	H31	Hybrid
P17	*P. pyrifolia*	H32	Hybrid
Red Hosui	*P. pyrifolia*	H33	Hybrid
		H34	Hybrid
		H35	Hybrid
		H36	Hybrid
		H37	Hybrid
		H38	Hybrid
		H40	Hybrid
		H41	Hybrid
		H42	Hybrid
		H45	Hybrid
		H46	Hybrid
		H47	Hybrid
		H48	Hybrid
		H49	Hybrid
		H50	Hybrid
		H51	Hybrid

The flavor volatile analysis procedure using GC-MS, was used as described by Rowan et al. (2009), except that fruit was placed in 4-L unused commercial metal paint cans rather than glass jars. Sample size varied from 300 to 1,000 g. Volatiles were collected onto Tenax-TA using an air flow of 55 ml/min for 2 h. After volatile collection, the absorbent traps were eluted with diethyl ether (2 × 1 mL) containing tetradecane at 10 nL mL^–1^ into pre-weighed 4 mL glass vials at a flow rate of 2 mL min^–1^. Samples were stored at −20°C before analysis using a Waters GCT GC-MS/Agilent 6890N GC equipped with an Optic 3 injector. Volatiles were identified based on their retention indices and by comparison with commercial mass spectral databases and authentic compounds. Generally, base peak intensities were used to aid automated peak identification and integration using Waters QuanLynx software. Fruit volume, and hence surface area, was calculated, and volatile concentrations are reported as ng tetradecane (*m*/*z* 57) equivalents released cm^–2^ fruit surface area per hour.

Young leaves were collected in spring 2013 for DNA extraction. Protocols for DNA extraction, genotyping-by-sequencing (GBS; Elshire et al., 2011) library preparation were the same as those reported earlier by Kumar et al. (2017). Briefly, GBS libraries were multiplexed into 5 pools, with 36–55 libraries per pool, for NGS sequencing on the Illumina HiSeq2000 platform and the sequence data were analyzed using TASSEL (Bradbury et al., 2007). The fastq file were mapped to the *P. × bretschneideri* (cultivar “Suli”) (Xue et al., 2018). SNPs with minor allele frequency (MAF) < 0.05, and missing data frequency > 20% were dropped.

### Measurement of ROH

**FIGURE 1:**
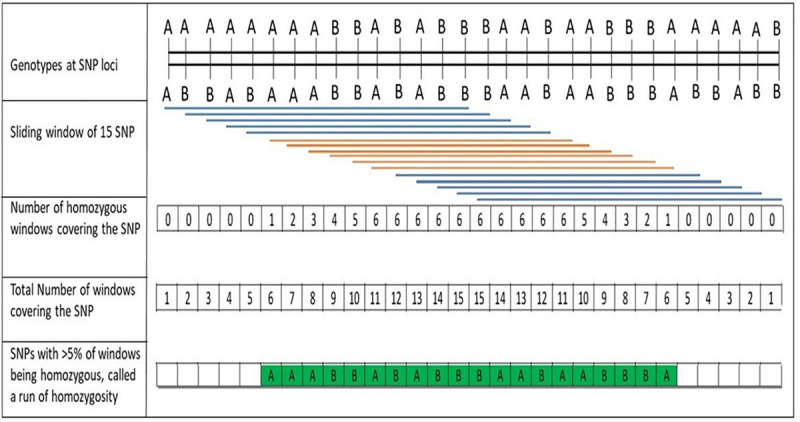
Description of the process for discovery of runs of homozygosity (ROH) using a sliding window of single nucleotide polymorphisms (SNPs) along the chromosome, as implemented with PLINK software (Purcell et al., 2007; Bjelland et al., 2013).

A procedure to discover ROH in PLINK software (Purcell et al., 2007) using a sliding-window approach along the genome is depicted in Figure 1. Briefly, a window of pre-determined number of SNPs was examined for homozygosity (allowing pre-determined number of heterozygous and missing calls) and then, for each SNP, the proportion of “homozygous” windows that overlap that position was calculated. ROH segments were then called based on a threshold for the average (Bjelland et al., 2013). To minimize the number of ROH that occurred by chance, the minimum number of SNPs that constituted a ROH was calculated following Lencz et al. (2007):

(1) $$l=\frac{\log_{e}\frac{\alpha }{n_{s}\,n_{i}}}{\log_{e}\left(1-h\,e\,t\right)}$$

where α is the percentage of false-positive ROH (set to 0.05), *n*_*s*_ is the number of SNPs per individual, *n*_*i*_ is the number of individuals and *het* is the mean SNP heterozygosity across all SNPs. Following Equation 1, the minimum number of SNPs constituting an ROH was set to 48 in this study. ROH segments were determined using PLINK v.1.7 based on the following settings: one heterozygous genotype and two missing SNP were allowed per window of 48 SNPs; minimum SNP density was set to one SNP per 50 kb, with a maximum gap between consecutive SNPs was set to 1 Mb to avoid low SNP density affecting ROH length; a minimum ROH length of 500 kb. The adjacent SNPs having a proportion of ROH occurrences over the adopted threshold formed ROH islands. In this study, putative ROH islands were determined based on overlapping ROH regions, shared by at least 15% of studied accessions. The adjacent SNPs were merged into genomic regions corresponding to ROH islands.

#### Measures of Genomic Inbreeding

For each accession, three estimates of the genomic inbreeding coefficient (F) were calculated, F_ROH_, F_SNP_, and F_GRM_. F_ROH_ is the fraction of each genome in ROH > 0.5 Mb. For example, in a sample for which *n* ROH of length *l*_*i*_ (in Mb) were identified, then F_ROH_ was calculated as:

(2) F=ROH1L∑inli

where L represents the genome length. F_SNP_, which is a method of moment based measure of inbreeding in the most recent generation (Clark et al., 2019), was estimated as follows using PLINK software:

(3) F=SNPO⁢(H⁢O⁢M)-E⁢(H⁢O⁢M)N-E⁢(H⁢O⁢M)

where O(HOM) is the observed number of homozygous SNPs, E(HOM) is the expected number of homozygous SNPs, and N is the total number of genotyped SNPs. F_GRM_, a genomic relationship-based inbreeding coefficient, was calculated using the method described by VanRaden et al. (2011):

(4) $$G=\frac{Z\,Z^{\prime }}{2\,\sum p\,\left(1-p\right)}$$

where ***Z*** is an *n* × *m* matrix (*n* = number of individuals, *m* = number of SNP loci) representing genotypes at each locus. The coefficient of the *i*th column of the ***Z*** matrix are (0–2*p*_*i*_), (1–2*p*_*i*_), and (2–2*p*_*i*_) for genotypes AA, AB, and BB, respectively, *p*_*i*_ is the allele frequency of allele A at the *i*th SNP. ***G*** was calculated using *p* = 0.5, which is the same as the method used by the USDA-ARS Animal Improvement Programs (VanRaden et al., 2011). The values on the diagonal of ***G*** denote the relationship of an accession to itself, or its genomic inbreeding coefficient (F_GRM_).

#### Effect Size Estimates for Quantitative Traits

For each trait, the phenotypes were modeled in two steps. First, a mixed linear model (MLM) was fitted accounting for fixed effects and random effects:

(5) y=X⁢b+Z⁢u+e

where y is a vector of measured trait values, *b* is a vector of unknown fixed covariate effects (e.g., overall mean, year effect), *X* and *Z* are the known design matrices for the fixed and random effects, respectively; *u* is an unknown vector of additive genetic effects with a normal distribution *N*(0, *σ*_*A*_^2^*G*), where *G* is the genomic relationship matrix (GRM); and *e* is an unknown vector of residuals. In the second step, estimates of random additive effects (*u*′) from Equation 5 were regressed on F_ROH_ as follows:

(6) $$u^{\prime }=\mu +\beta *F_{\text{ROH}}+\varepsilon$$

where μ is the overall mean, β is the unknown scalar effect of F_ROH_ on the trait, F_ROH_ is a known vector of individual F_ROH_, and ε is an unknown vector of residuals.

Marker-trait genome-wide association (GWA) analysis were also conducted for each trait using unified MLM as implemented in R package GAPIT (Lipka et al., 2012). Principal components (PCs) analysis was used to quantify patterns of population structure, and the first two PCs were used as covariate to avoid spurious marker-trait associations that could arise from population structure. Co-localization of ROH islands with trait-associated SNPs, and overlap with previously reported QTLs (reviewed by De Franceschi and Dondini, 2019), was also investigated.

## Results

### ROH in Different Genetic Groups

**FIGURE 2:**
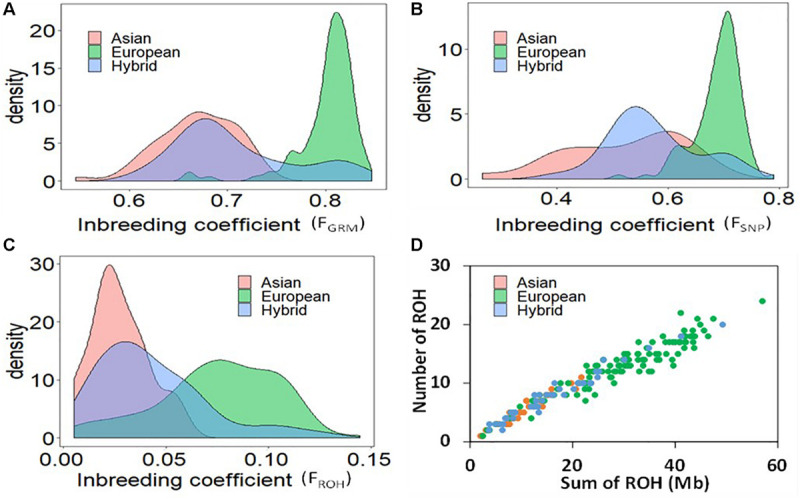
Distribution of the measures of genomic inbreeding coefficients based on genomic relationship matrix **(A)**, Method of moments **(B)**, and runs of homozygosity **(C)**; and the relationship between the total genomic length covered by ROH and the total number of ROH per individual **(D)** in Asian, European and hybrid pear population.

After quality controls (i.e., missing data frequency < 20%, minor allele frequency > 0.05), about 8500 SNPs distributed across the genome (Supplementary Figure S1) were retained for further analysis. The first principal component (PC1) grouped the Asian and European accessions in two non-overlapping clusters (Supplementary Figure S2). The hybrids resided in between the two main clusters, but many hybrid accessions grouped closely with either Asian or European species. Figures 2A–C displays the distributions of F_GRM_, F_SNP_, and F_ROH_, respectively, with means of 0.52, 0.67, and 0.03 in Asian accessions; 0.69, 0.80, and 0.08 in European accessions; and 0.57, 0.71, 0.04 in the hybrid population. The accessions with smaller F_ROH_ were considered as the least inbred, whereas accessions with larger F_ROH_ were considered as the most inbred accessions. Correlations between the three measures of genomic inbreeding were large, with correlations between F_SNP_ and F_ROH_ of 0.64, F_SNP_ and F_GRM_ of 0.94, and F_ROH_ and F_GRM_ of 0.74.

The relationship between the total genomic length (Mb) covered by ROH and the total number of ROH per accession demonstrates separation between the European and Asian pear species (Figure 2D). The average number of ROH per accession was 6, 13 and 8 for Asian, European and hybrid accessions, respectively. The average total genomic length of ROH was 11, 30 and 17 Mb for Asian, European and hybrids accessions, respectively. One accession of European pear (“Nellie”) presented total ROH length of about 60 Mb. The average length of ROH in Asian, European and hybrid accessions was 1.96, 2.29, and 2.08 Mb, respectively. The occurrence of ROH on different linkage groups was generally similar between the two species, but a much higher frequency was observed in Asian accessions on LG4 (12 vs. 4.8%) and LG17 (13.2 vs. 8.7%), while European accessions displayed a higher proportion of ROH on LG13 (6.4 vs. 2.8%) and LG15 (11.4 vs. 4.5%) (Supplementary Figure S3).

**FIGURE 3:**
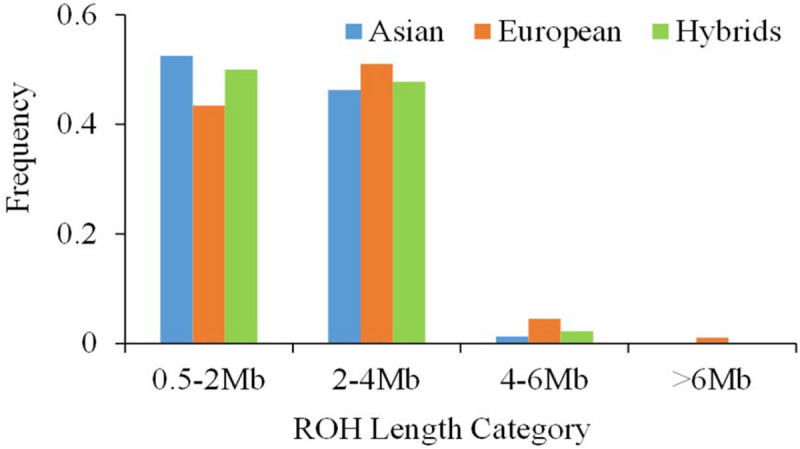
The distribution of the single runs of homozygosity (ROH) length classes within the Asian, European and hybrid pear (*Pyrus* spp.) populations.

Classification of ROH by length showed that the majority of ROHs were shorter than 4 Mb in all three pear genetic groups (Figure 3). For ROHs shorter than 2 Mb, Asian and European accessions had the highest (ca. 50%) and the lowest (ca. 40%) proportions, respectively. ROH segments 4–6 Mb long were more frequent in European (5%) accessions compared with the Asians (1%). None of the ROH in Asian and hybrid accessions were longer than 6 Mb, but the European accessions showed few ROH close to 10 Mb length.

#### Trait-F_ROH_ Associations

**FIGURE 4:**
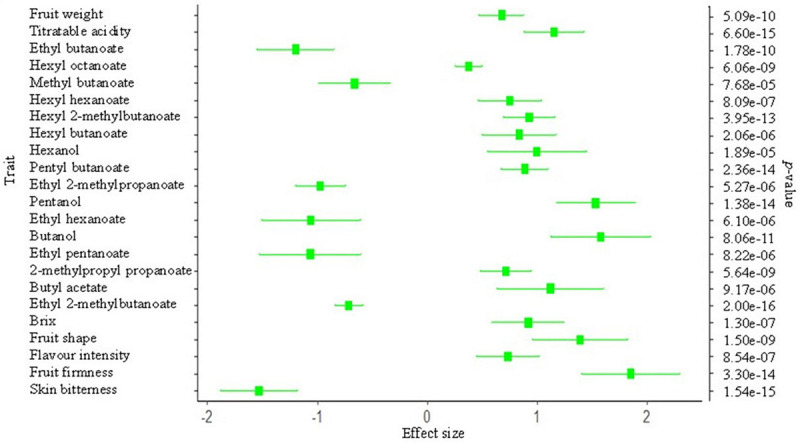
Effect of inbreeding (F_ROH_)/selection on various pear (*Pyrus* spp.) phenotypes. The x-axis represents the effect size (in phenotypic standard deviation unit) estimate of F_ROH_ = 0.15. The secondary *y*-axis shows the probability of significance of the effect size for traits that reached Bonferroni-corrected significance of probability value (=0.01/32 traits).

Trait values were regressed on F_ROH_ to estimate the effect of inbreeding/selection on each of the 32 traits considered in this study, with 23 reaching experiment-wise significance threshold (0.01/32 = *p* < 3.1e-04). Effect size, in phenotypic standard deviation units (*σ_*p*_*), corresponding to F_ROH_ = 0.15 (equivalent to the maximum value observed in this study) are shown in Figure 4. An increase of 0.15 in F_ROH_ was associated with 0.70*σ_*p*_* and 1.8*σ_*p*_* increase in fruit weight and fruit firmness, respectively. Non-volatile compounds (Brix and TA), which partly influence sensory flavor intensity, increased by about 1.0*σ_*p*_* at F_ROH_ = 0.15. Volatile compounds, alcohols and non-ethyl esters (esters not derived from esterification with ethanol), increased with increasing F_ROH_. Skin bitterness and ethyl esters decreased significantly with increases in F_*RO*__*H*_ (Figure 4).

### Islands of ROH

**FIGURE 5:**
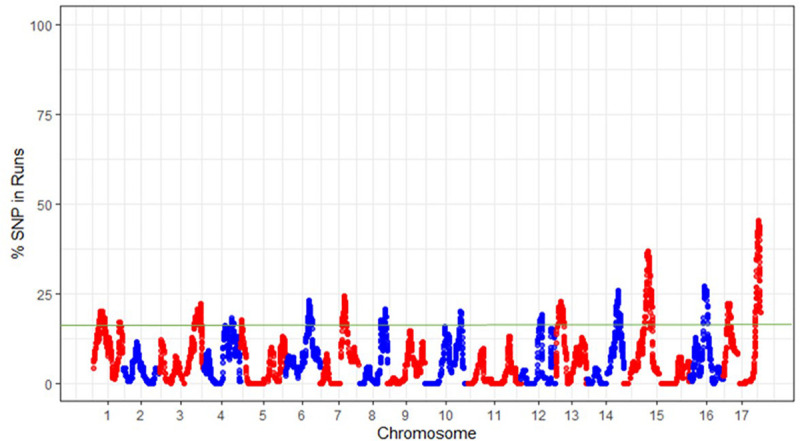
The frequency (%) of single nucleotide polymorphisms (SNPs) occurrence into runs of homozygosity (ROH) islands across the pear (*Pyrus* spp.) genome. The green horizontal line indicates the adopted threshold (15%), which defines the ROH islands.

In the significant ROH islands described here using combined samples from all three groups, each SNP showed a percentage of occurrence > 15% (Figure 5). This approach resulted in the identification of 20 ROH islands, with a maximum of two ROH islands on some linkage groups (e.g., LGs 1, 4, 8, 10, 12, and 17) and no significant island on LGs 2, 9, and 11. The smallest and the longest significant ROH island were observed on LG5 (0.770–0.772 Mb) and LG15 (12.718–17.131 Mb), respectively (Supplementary Table S1). Within the ROH island on LG15, a homozygous haplotype (GCGAAT) comprising six SNPs spanning over a 71 bp region (14,017,541–14,017,612 bp) was shared by 48, 91, and 90% accessions of Asian, European and hybrid populations, respectively. The occurrence of ROH islands was also investigated in each genetic group separately, which revealed some key differences between these groups (Supplementary Figure S4).

**FIGURE 6:**
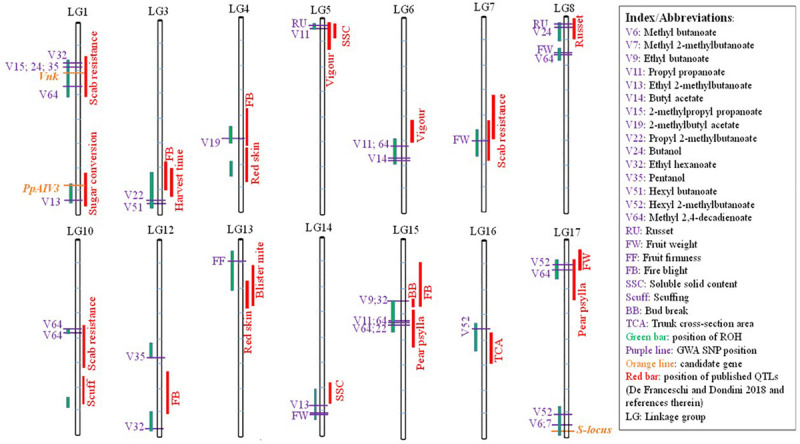
Co-localization of runs of homozygosity islands (green bars), earlier published QTLs for various traits (red bars), marker-trait associations (purple horizontal lines) and candidates genes (orange horizontal lines).

A search for co-localization of ROH islands with previously published QTLs revealed that 18 out of 20 ROH islands overlapped with QTLs for various traits (Figure 6). In addition to numerous QTLs, candidate genes *Vnk* (which confer resistance to *Venturia nashicola*) and *PpAIV3* (controlling conversion of sucrose to hexose in mature fruit) resided within ROH islands on LG1. A 3.2 Mb long ROH island at the bottom of LG17 overlapped with the self-incompatibility gene (*SI-locus*). Using conventional GWA, with a genome-wide significance threshold of *p* < 0.05, a total of 294 SNPs were found to be significantly associated with at least one of the 33 traits considered in this study. Thirty-seven out of 294 significant SNPs resided within the significant ROH islands (Figure 6; Supplementary Table S2). A SNP associated with skin russet resided within an ROH island in the upper region of LG8, which has previously been reported a harboring a QTL for this trait.

## Discussion

The intense selection in commercial species has necessitated strategies to characterize and monitor inbreeding and maintain genetic diversity in long-term breeding and conservation programs (De Cara et al., 2013; Bosse et al., 2015). The absence of pedigree information on wild and semi-wild accessions makes it difficult to estimate observed levels of inbreeding in the current germplasm resources of fruit crops. Results from studies on livestock species (Toro and Varona, 2010; Bjelland et al., 2013; Peripolli et al., 2017) have shown that using genomic inbreeding estimates (F_ROH_) improves mating decisions and gene conservation efforts. The average inbreeding coefficient (F_ROH_) in the inter-specific hybrid population was similar to the Asian accessions, but lower compared with the European accessions. The first generation (F_1_) hybrid population is expected to display lower F_ROH_ than the parental species. Most of the hybrids used in this study were second-generation accessions selected from the F_2_ or BC1 crosses. The clustering patterns from PCA analysis also showed greater resemblance of many hybrid accessions with either the Asian or European species. Therefore, the artificial selection and the mating scheme in the inter-specific breeding program would have led to the observed higher level of F_ROH_ in the studied hybrid accessions. Use of genome sequence information has been advocated for monitoring and utilization of gene-banks of plant species (Henry, 2012; Mascher et al., 2019). Results from our study suggested that F_ROH_ derived from using a high-density genotyping platform would provide a novel tool for managing diversity of plant genetic resources.

### Assessment of Runs of Homozygosity

In our study, we used a window size of 48 SNPs to identify ROH in *Pyrus* spp. Studies on dairy cattle have shown that for a window size of 20–50 SNPs, F_ROH_ was more accurate than F_GRM_ derived from the observed genomic relationship matrices (Kim et al., 2013; Forutan et al., 2018). The correlation between F_ROH_ and F_GRM_ was high (0.74), but the substantially higher value of F_GRM_ compared to F_ROH_ (Figure 2) is likely due to the fact that the base allele frequencies were not known and F_GRM_ cannot distinguish between alleles that are identity-by-descent and identity-by-state. Very similar correlations were observed between F_ROH_ and F_GRM_ in studies on dairy cattle (Bjelland et al., 2013; Forutan et al., 2018). In our study, the average F_ROH_ in European accessions (0.08) was higher compared with that of the Asian (0.03) accessions. There are no reports of F_ROH_ in self-incompatible plant species, but the much higher F_ROH_ (often > 0.15) for livestock species reflects stronger artificial selection (review by Peripolli et al., 2017) compared with plant species investigated in this study.

Different mating systems, selection directions and population development histories will form unique ROH distribution patterns in the genomes of geographically isolated populations (Bosse et al., 2012). Our results showed that the number and total length of ROH in Asian accessions were shorter than in European accessions, and longer ROH segments (>2 Mb) were more frequent in European accessions; suggesting relatively lower nucleotide diversity in European pear. Using whole genome re-sequencing, Wu et al. (2018) observed that Asian pears had a higher nucleotide diversity than European pears, which supports ROH patterns observed in this study. Compared with the Asian group, the European accessions clearly displayed higher individual sums of ROH per accession (Figure 2). Taken together, these results suggest that systematic breeding of European pears would have started earlier than Asian pears. The pattern of ROH in the European pears could also be a result of population bottlenecks due to glaciation in Europe as compared to Asia. Similar inferences were drawn when ROH patterns were compared between Asian and European livestock populations (Groenen et al., 2012; Peripolli et al., 2017).

Further investigation of ROH islands showed that generally different haplotypes were observed in accessions of the two species. The longest (71 bp) haplotype shared by both the Asian and European species resided on LG15. The small size of the shared ROH haplotype would indicate these two species shared ancestry many thousands of generations ago. Assuming an average genetic map size 1,350 cM and a genome size of 527 Mb (Li et al., 2017; Xue et al., 2018), one Mb physical distance approximately equates to 2.5 cM genetic distance in *Pyrus*. So, an ROH of length 0.0001775 cM would have arisen from a common ancestor occurring approximately 282,000 generations ago (*g*=100/(2×0.0001775); Fisher, 1954). Assuming a generation interval of 10 years in *Pyrus*, this would suggest that Asian and European species would have shared common ancestors at least 2.8 million years ago, which is supported by a study on the domestication history of pears (Wu et al., 2018).

### Association Between ROH and Phenotypes

Genomic regions that are selection targets tend to generate ROH islands around the selected locus compared to the rest of the genome. Based on the observed similarity of nucleotide diversity between wild and cultivated pears, Wu et al. (2018) suggested relatively weak selection during pear domestication—which is supported by our observations of relatively low F_ROH_ compared with commercial livestock species (Peripolli et al., 2017). The ROH patterns in Asian and European populations showed some major differences on some linkage groups (Supplementary Figures S3, S4), which are supported by previous analysis of selective sweeps showing that genomic regions were differentially selected between Asian and European pears for traits such as cell wall degradation, fruit size, sugar biosynthesis, stone cells, acid and volatile compounds (Wu et al., 2018). ROH size and frequency were also reported to vary between Asian and European livestock populations (Bosse et al., 2012; Groenen et al., 2012; Peripolli et al., 2017).

Significant effects of increase in homozygosity were observed on various fruit phenotypes in this study. Increasing F_ROH_ significantly increased various traits such as fruit firmness, Brix, fruit weight, sensory flavor intensity, and TA. We also found strong evidence (*P* < 1 × 10^–6^) of negative selection for traits, including skin bitterness and ethyl esters, with an increase in F_ROH_. Flavor volatiles have not directly been the target of artificial selection in pear breeding (Brewer and Palmer, 2010), however, these phenotypes are indirectly influenced by selection for other traits such as sensory flavor. Interestingly, ethyl esters were adversely correlated with sensory flavor intensity, suggesting that these particular esters are not important contributors to flavor intensity. Non-ethyl esters and the alcohols (hexanol, pentanol, and butanol) showed signatures of positive selection, which could largely be due to their favorable association with the breeding target traits (e.g., soluble solids and sensory flavor).

### ROH Islands and Candidate Genes

The genomic regions with high occurrence of ROH have been shown to contain important genes associated with phenotypes in humans (Ceballos et al., 2018; Clark et al., 2019) and livestock species (Purfield et al., 2012; Kim et al., 2013; Biscarini et al., 2014). Overlap of ROH islands with marker-trait associations identified in this study, as well as with the previously published QTLs for pear fruit/tree phenotypes (De Franceschi and Dondini, 2019; Kumar et al., 2019), adds to the robustness of ROH mapping as a complementary strategy for GWA studies in outbred fruit crops.

An ROH island at the bottom of LG17 harbors the self-incompatibility (SI) gene family which includes S-RNase and S-locus F-Box Brothers (SFBB) genes (Yamamoto et al., 2002; De Franceschi et al., 2011). It has been suggested that in addition to artificial selection, ROH islands could also be an indication of a lower recombination rate in those regions (Peripolli et al., 2017), which makes perfect sense in the case of the *Pyrus* SI-locus. Recombination suppression in the SI-locus region is essential because the pistil and pollen genes must inherit as one single unit in order to maintain the functionality of the SI system (Roalson and McCubbin, 2003; Claessen et al., 2019). Wu et al. (2013) reported highly repetitive sequences in the SI-locus region of pear, and hypothesized that suppression of recombination in the SI-locus region may be related to the presence of many repetitive sequences. Recombination between the pistil-*S* and pollen-*S* determinant genes would result in non-functional *S*-haplotypes and loss of self-incompatibility. The recombination suppressed region in *Pyrus* is predicted to be much larger compared to some other fruit species (Matsumoto and Tao, 2016).

Selection would result in selective sweeps, which refers to the genomic regions that have reduced nucleotide diversity compared with randomly evolving regions. Sabeti et al. (2002) developed the extended haplotype homozygosity (EHH) method to identify selective sweep regions in the human genome, and this tool has also been used to detect population-specific signatures of selection in livestock populations (Qanbari et al., 2010; Bomba et al., 2015). Short ROH regions were shown to overlap with EHH regions, suggesting complimentary nature of these two approaches to identify genomic regions under selection (Zhang et al., 2015). However, ROH patterns provide a guide to the population history (demography) and selection, which makes it a powerful tool for management of plant genetic resources, as well as for trait architecture studies in self-incompatible plants.

A key strength of ROH mapping is that long homozygous segments of genomes can be reliably identified using relatively low marker densities. However, the reduced-representation low-coverage genotyping platform used in our study could have missed many shorter ROH, which would result in an underestimation of F_ROH_. Studies on humans (Ceballos et al., 2018) and livestock species (Purfield et al., 2012) have shown that high-density genotyping would be desirable, especially for mapping of shorter ROH. Further studies using the recently developed 200K SNP array (Li et al., 2019) or whole genome sequence data should help map shorter ROH for more accurate estimate of F_ROH_ and enable us to detect many more signals of natural and/or artificial selection in *Pyrus*.

## Conclusion

In summary, this first application of the ROH approach in self-incompatible fruit crop species enabled us to compare genomic inbreeding coefficients between *Pyrus* species differing in domestication and breeding histories. For outbred fruit crops, genomic measure of inbreeding (F_ROH_) would serve as a novel tool for breeding and management of gene-banks lacking reliable pedigree information. Association between F_ROH_ and phenotypes provides a simple mechanism to evaluate the direction of phenotypic change because of increased inbreeding levels. Co-localization of ROH islands and GWA signals agreed with results from studies in humans and livestock populations, which suggested that ROH mapping offers a complementary strategy to understand the genetic architecture of complex traits. Distribution of ROH islands in different species or populations of fruit crops can effectively be used to evaluate signatures of differential selection.

## Data Availability Statement

The genotyping-by-sequencing data presented in the study are deposited in the https://zenodo.org/ repository, digital object identifier https://zenodo.org/record/4302655, https://zenodo.org/record/4308154, and https://zenodo.org/record/4304904.

## Author Contributions

SK conceived the study, conducted ROH and GWA analyses, and wrote the first draft. SK and LB designed the study. CW and CK led leaf collection, DNA extraction, and GBS library preparation. CD conducted bioinformatics analysis. JW conducted QTL and candidate gene searches. DR, MH, and LB led the phenotyping. All authors helped to edit the manuscript, and read and approved the manuscript.

## Conflict of Interest

The authors declare that the research was conducted in the absence of any commercial or financial relationships that could be construed as a potential conflict of interest.

## Supplementary Material

Supplementary Figure S1 Distribution of about 8500 single nucleotide polymorphism (SNP) markers on *Pyrus bretschneideri* “DangshanSuli”v1. 0 genome. The *x*-axis indicates linkage group number and *y*-axis length in base-pairs.

Supplementary Figure S2 Population structure of Asian, European and inter-specific hybrid pear accessions using principal components analysis (PCA).

Supplementary Figure S3 Distribution of the runs of homozygosity (ROH) across different linkage groups in Asian, European and Hybrid pear (*Pyrus* spp.) accessions.

Supplementary Figure S4 The frequency (%) of single nucleotide polymorphisms (SNPs) occurrence into runs of homozygosity (ROH) islands within the Asian, European and hybrid pear (*Pyrus* spp.) population. The blue and red color represent chromosomes.

Supplementary Table S1 The list of genomic regions of extended homozygosity (runs of homozygosity (ROH) islands). The overlap between genomic locations of ROH islands and previously published quantitative trait loci (QTLs) are approximated based on a recent review published by De Franceschi and Dondini (2019).

Supplementary Table S2 Genomic positions of trait-associated single nucleotide polymorphisms (SNPs) which resided within the runs of homozygosity (ROH) islands on different linkage groups (LG). The probability of significance and the minor allele frequency (MAF) at each SNP loci is also shown.
