# Supplementary material for: Homozygosity Mapping Reveals Population History and Trait Architecture in Self-Incompatible Pear (Pyrus spp.)
Source: Front Plant Sci. 2021 Jan 5;11:590846. doi: 10.3389/fpls.2020.590846 (PMC7813798; doi:10.3389/fpls.2020.590846)
Supplement: Supplementary Figure S1 — Distribution of about 8500 single nucleotide polymorphism (SNP) markers on Pyrus bretschneideri “DangshanSuli”v1. 0 genome. The x-axis indicates linkage group number and y-axis length in base-pairs. [file Data_Sheet_1.PDF]

Supplementary Table S1. The list of genomic regions of extended homozygosity (runs of homozygosity (ROH) islands). The overlap between genomic locations of ROH islands and previously published quantitative trait loci (QTLs) are approximated based on a recent review published by De Franceschi and Dondini (2019)[#].

Linkage group	Number of SNPs	Start bp	End bp	Length (bp)	QTL/Genes (Reference)[#]
1	90	3,156,107	6,784,990	3,628,883	Scab resistance (Perchepied et al. 2015); *Vnk* gene (Terakami et al. 2006)
1	22	15,221,214	16,494,877	1,273,663	Sucrose conversion (Nishio et al. 2018); *PpAIV3* (Nishio et al. 2018)
3	156	21,296,184	25,685,409	4,389,225	Fire blight (Bokszczanin et al. 2011); Harvest time (Yamamoto et al. 2014)
4	7	12,965,813	14,131,220	1,165,407	Fire blight (Dondini et al. 2004);
4	34	16,824,577	18,059,342	1,234,765	Red skin (Dondini et al. 2008; Kumar et al. 2019a)
5	23	770,408	772,133	1,725	SSC (Wu et al. 2014); Vigour (Knabel et al. 2015)
6	105	13,134,381	15,901,406	2,767,025	Vigour (Knabel et al. 2015)
7	93	13,285,411	16,086,266	2,800,855	Scab resistance (Pierantoni et al. 2007; Won et al. 2014)
8	51	923,873	2,312,707	1,388,870	Russet (Yamamoto et al. 2014)
8	36	3,180,786	3,888,602	707,816	
10	19	11,828,431	12,040,190	211,759	Scab resistance (Won et al. 2014)
10	42	20,964,170	22,188,417	1,224,247	Scuffing (Kumar et al. 2019b)
12	43	11,135,206	12,444,832	1,309,626	
12	39	18,071,226	19,446,996	1,375,770	Fire blight (Montanari et al. 2016)
13	102	1,159,166	4,971,121	3,811,955	Red skin (Wu et al. 2014); Blister mite (Brewer et al. 2018)
14	136	16,815,038	19,888,362	3,073,324	Soluble solid content (Wu et al. 2014)
15	150	12,718,895	17,131,809	4,412,914	Bud break (Gabay et al. 2018); Fire blight (Montanari et al. 2015); pear psylla (Montanari et al. 2016)
16	68	8,461,376	10,986,781	2,525,405	Rootstock trunk cross-sectional area (Knabel et al. 2015)
17	50	2,766,324	4,612,441	1,846,117	Fruit weight (Wu et al. 2014); Pear psylla (Dondini et al. 2015)
17	112	19,397,320	22,602,851	3,205,531	Self-incompatibility locus (Yamamoto et al. 2004; De Franceschi et al. 2011)

Supplementary Table S1 continued

[#]References

Bokszczanin KL, Przybyla AA, Dondini L, Palucha A (2011) QTLs for fire blight (Erwinia amylovora) resistance in Pyrus ussuriensis. Acta Hortic 371–373.

Brewer L, Shaw P, Wallis R, Alspach P, Aldworth M, Orellana-torrejon C, Chagné D, Bus VGM (2018) Genetic mapping of pear sawfly (Caliroa cerasi) and pear blister mite (Eriophyes pyri) resistance in an interspecific pear family. *Tree Genetics & Genomes* **14**: 38.

De Franceschi P, Pierantoni L, Dondini L, Grandi M, Sanzol J, Sansavini S (2011) Cloning and mapping multiple S-locus F-box genes in European pear (Pyrus communis L.). *Tree Genetics & Genomes* **7**: 231–240

De Franceschi P, Dondini L (2019) Molecular Mapping of Major Genes and QTLs in Pear. In *The Pear Genome* (Korban SS, ed), pp 113-131. Springer Cham.

Dondini L, Pierantoni L, Gaiotti F, Chiodini R, Tartarini S, Bazzi C, Sansavini S (2004) Identifying QTLs for fire-blight resistance via a European pear (Pyrus communis L.) genetic linkage map. *Molecular Breeding* **14**: 407–418.

Dondini L, Pierantoni L, Ancarani V, D'Angelo M, Cho KH, Shin IS, Musacchi S, Kang SJ, Sansavini S (2008) The inheritance of the red colour character in European pear (Pyrus communis) and its map position in the mutated cultivar "Max Red Bartlett". *Plant Breeding* **127**: 524–526.

Dondini L, De Franceschi P, Ancarani V, Civolani S, Fano EA, Musacchi S (2015) Identification of a QTL for psylla resistance in pear via genome scanning approach. *Scientia Horticulturae* **197**: 568–572.

Gabay G, Dahan Y, Izhaki Y, Faigenboim A, Ben-Ari G, Elkind Y, Flaishman MA. (2018) High-resolution genetic linkage map of European pear (Pyrus communis) and QTL fine-mapping of vegetative budbreak time. *BMC Plant Biology* **18**: 175.

Knäbel M, Friend AP, Palmer JW, Diack R, Wiedow C, Alspach P, Deng C, Gardiner SE, Tustin DS, Schaffer, R, Foster T, Chagné D (2015) Genetic control of pear rootstock-induced dwarfing and precocity is linked to a chromosomal region syntenic to the apple Dw1 loci. *BMC Plant Biology* **15**: 230.

Kumar S, Kirk C, Deng CH, Wiedow C, Qin M, Espley R, Wu J, Brewer L. (2019a) Fine-mapping and validation of the genomic region underpinning pear red skin colour. *Horticulture Research* **6**: 1-7.

Kumar S, Kirk C, Deng CH, Shirtliff A, Wiedow C, Qin M, Wu J, Brewer L. (2019b) Marker-trait associations and genomic predictions of interspecific pear (Pyrus) fruit characteristics. *Scientific Reports* **9**: 1-10.

Montanari S, Guérif P, Ravon E, Denancé C, Muranty H, Velasco R, Chagné D, Bus VGM, Robert P, Perchepied L, Durel CE (2015) Genetic mapping of Cacopsylla pyri resistance in an interspecific pear (Pyrus spp.) population. *Tree Genetics & Genomes* **11**: 74

Montanari S, Perchepied L, Renault D, Frijters L, Velasco R, Horner M, Gardiner SE, Chagné D, Bus VGM, Durel CE, Malnoy M (2016) A QTL detected in an interspecific pear population confers stable fire blight resistance across different environments and genetic backgrounds. *Molecular Breeding* **36**: 47.

Nishio S, Saito T, Terakami S, Takada N, Kato H, Itai A (2018) Identification of QTLs Associated with conversion of sucrose to hexose in mature fruit of Japanese pear. *Plant Molecular Biology Reporter* **36**: 643–652

Perchepied L, Leforestier D, Ravon E, Guérif P, Denancé C, Tellier M, Terakami S, Yamamoto T, Chevalier M, Lespinasse Y, Durel CE (2015) Genetic mapping and pyramiding of two new pear scab resistance QTLs. *Molecular Breeding* **35**: 197.

Pierantoni L, Dondini L, Cho KH, Shin IS, Gennari F, Chiodini R, Tartarini S, Kang SJ, Sansavini S (2007) Pear scab resistance QTLs via a European pear (Pyrus communis) linkage map. *Tree Genetics & Genomes* **3**: 311-317.

Terakami S, Shoda M, Adachi Y, Gonai T, Kasumi M, Sawamura Y, Iketani H, Kotobuki K, Patocchi A, Gessler C, Hayashi T, Yamamoto T (2006) Genetic mapping of the pear scab resistance gene Vnk of Japanese pear cultivar Kinchaku. *Theoretical & Applied Genetics* **113**: 743–752.
Won K, Bastiaanse H, Kim YK, Song JH, Kang SS, Lee HC, Cho KH, Brewer L, Singla G, Gardiner SE, Chagné D, Bus VGM (2014) Genetic mapping of polygenic scab (Venturia pirina) resistance in an interspecific pear family. *Molecular Breeding* **34**: 2179–2189
Wu J, Li LT, Li M, Khan MA, Li XG, Chen H, Yin H, Zhang SL (2014a) High-density genetic linkage map construction and identification of fruit-related QTLs in pear using SNP and SSR markers. *Journal of Experimental Botany* **65**: 5771–5781.
Yamamoto T, Kimura T, Saito T, Kotobuki K, Matsuta N, Liebhard R, Gessler C, van de Weg WE, Hayashi T (2004) Genetic linkage maps of Japanese and European pears aligned to the apple consensus map. *Acta Horticulturae* **663**: 51-56.
Yamamoto et al. 2014. Yamamoto T, Terakami S, Takada N, Nishio S, Onoue N, Nishitani C, Kunihisa M, Inoue E, Iwata H, Hayashi T, Itai A, Saito T (2014) Identification of QTLs controlling harvest time and fruit skin color in Japanese pear (Pyrus pyrifolia Nakai). *Breeding Science* **64**: 351–361

Supplementary Table S2. Genomic positions of trait-associated single nucleotide polymorphisms (SNPs) which resided within the runs of homozygosity (ROH) islands on different linkage groups (LG). The probability of significance and the minor allele frequency (MAF) at each SNP loci is also shown.

LG	Position	Trait	*p-value*	MAF
1	3277864	Ethyl hexanoate	6.00E-06	0.22
1	4085440	2-methylpropyl propanoate; Butanol; Pentanol	1.21E-06	0.43
1	5896015	Methyl 2,4-decadienoate	2.36E-06	0.12
1	16494842	Ethyl 2-methylbutanoate	5.20E-09	0.11
3	25194167	Propyl 2-methylbutanoate	5.98E-06	0.05
3	25385546	Hexyl butanoate	3.79E-06	0.30
4	14131220	2-methylbutyl acetate	4.55E-06	0.24
5	770505	Russet	1.89E-07	0.16
5	770723	Propyl propanoate	1.72E-06	0.10
6	13981778	Propyl propanoate; Methyl 2,4-decadienoate	2.98E-07	0.46
6	15679252	Butyl acetate	3.09E-06	0.42
6	15679263	Butyl acetate	2.54E-06	0.38
7	14497005	Fruit weight	2.32E-06	0.24
8	923827	Russet	1.08E-06	0.09
8	1005084	Butanol	3.38E-06	0.31
8	3604897	Fruit weight	1.54E-06	0.45
8	3604912	Methyl 2,4-decadienoate	2.06E-06	0.14
8	3604919	Methyl 2,4-decadienoate	6.70E-07	0.09
6	16293177	Russet	1.08E-06	0.09
10	11828431	Methyl 2,4-decadienoate	2.16E-06	0.33
10	12040167	Methyl 2,4-decadienoate	2.00E-06	0.14
12	12444832	Pentanol	1.88E-06	0.23
12	19446867	Ethyl hexanoate	5.18E-06	0.31
13	2391902	Fruit firmness	1.97E-06	0.07
14	18760535	Ethyl 2-methylbutanoate	3.70E-06	0.06
14	19719328	Fruit weight	1.59E-06	0.31
14	19719381	Fruit weight	3.33E-07	0.32
15	12785106	Ethyl butanoate; Ethyl hexanoate	1.65E-06	0.08
15	16134909	Propyl propanoate; Methyl 2,4-decadienoate	1.96E-06	0.14
15	16134956	Propyl propanoate; Methyl 2,4-decadienoate	1.42E-06	0.14
15	16135002	Propyl propanoate; Methyl 2,4-decadienoate; Propyl 2-methylbutanoate	1.41E-06	0.19
15	16135045	Propyl 2-methylbutanoate	7.31E-07	0.14
16	9176161	Hexyl 2-methylbutanoate	1.22E-07	0.38
17	3127713	Hexyl 2-methylbutanoate	1.09E-06	0.17
17	3675341	Methyl 2,4-decadienoate	2.39E-06	0.39
17	20213066	Hexyl 2-methylbutanoate	2.14E-06	0.37
17	21693476	Methyl butanoate; Methyl 2-methylbutanoate	2.39E-06	0.27

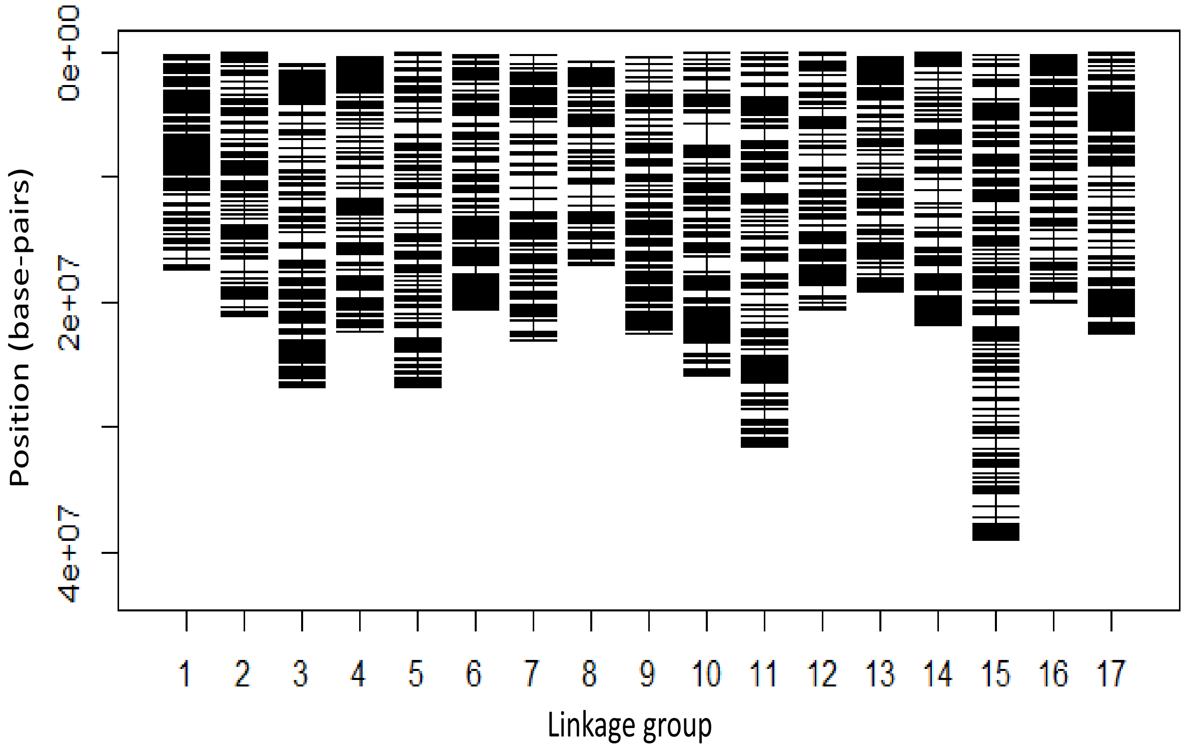

Supplementary Figure S1. Distribution of about 8500 single nucleotide polymorphism (SNP) markers on *Pyrus bretschneideri* 'DangshanSuli'v1. 0 genome. The x-axis indicates linkage group number and y-axis length in base-pairs.

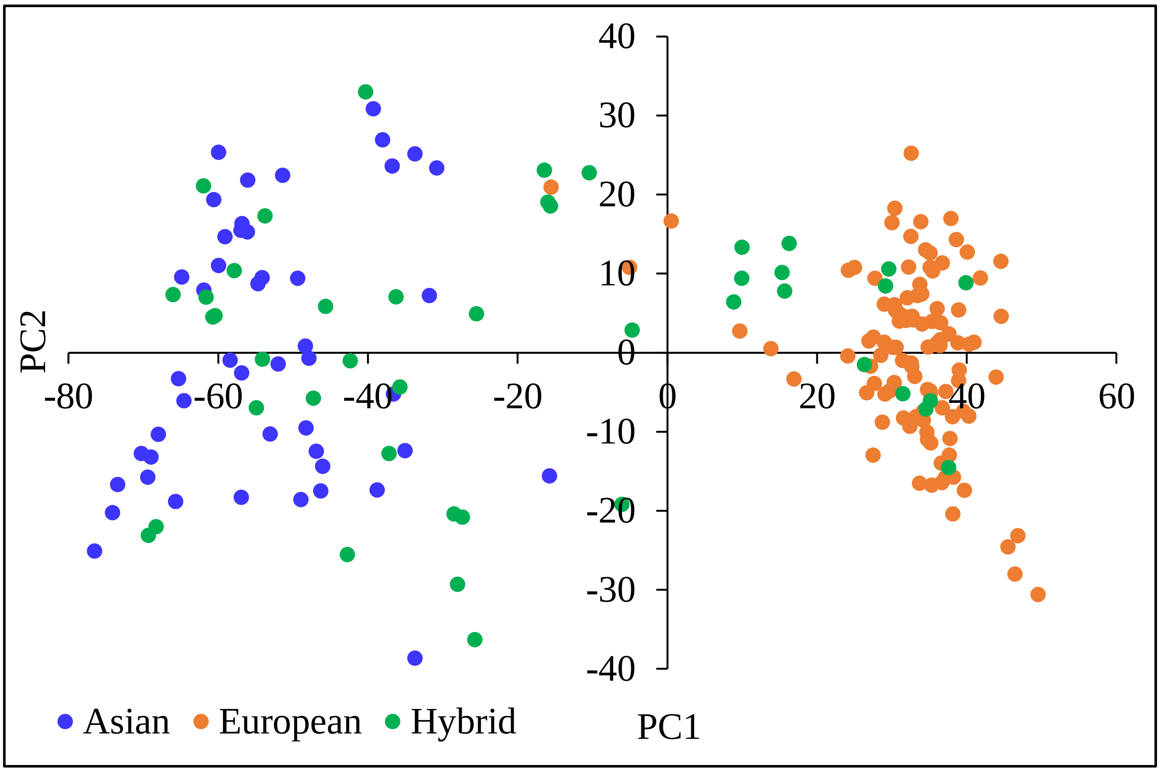

Supplementary Figure S2. Population structure of Asian, European and inter-specific hybrid pear accessions using principal components analysis (PCA).

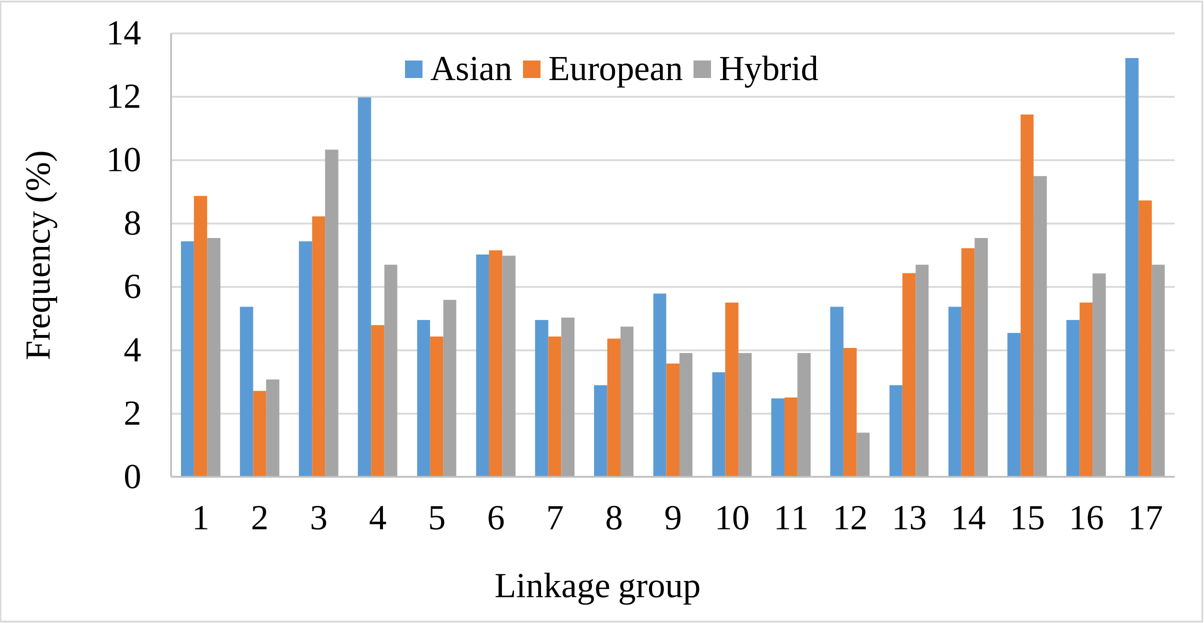

Supplementary Figure S3. Distribution of the runs of homozygosity (ROH) across different linkage groups in Asian, European and Hybrid pear (*Pyrus* spp.) accessions.

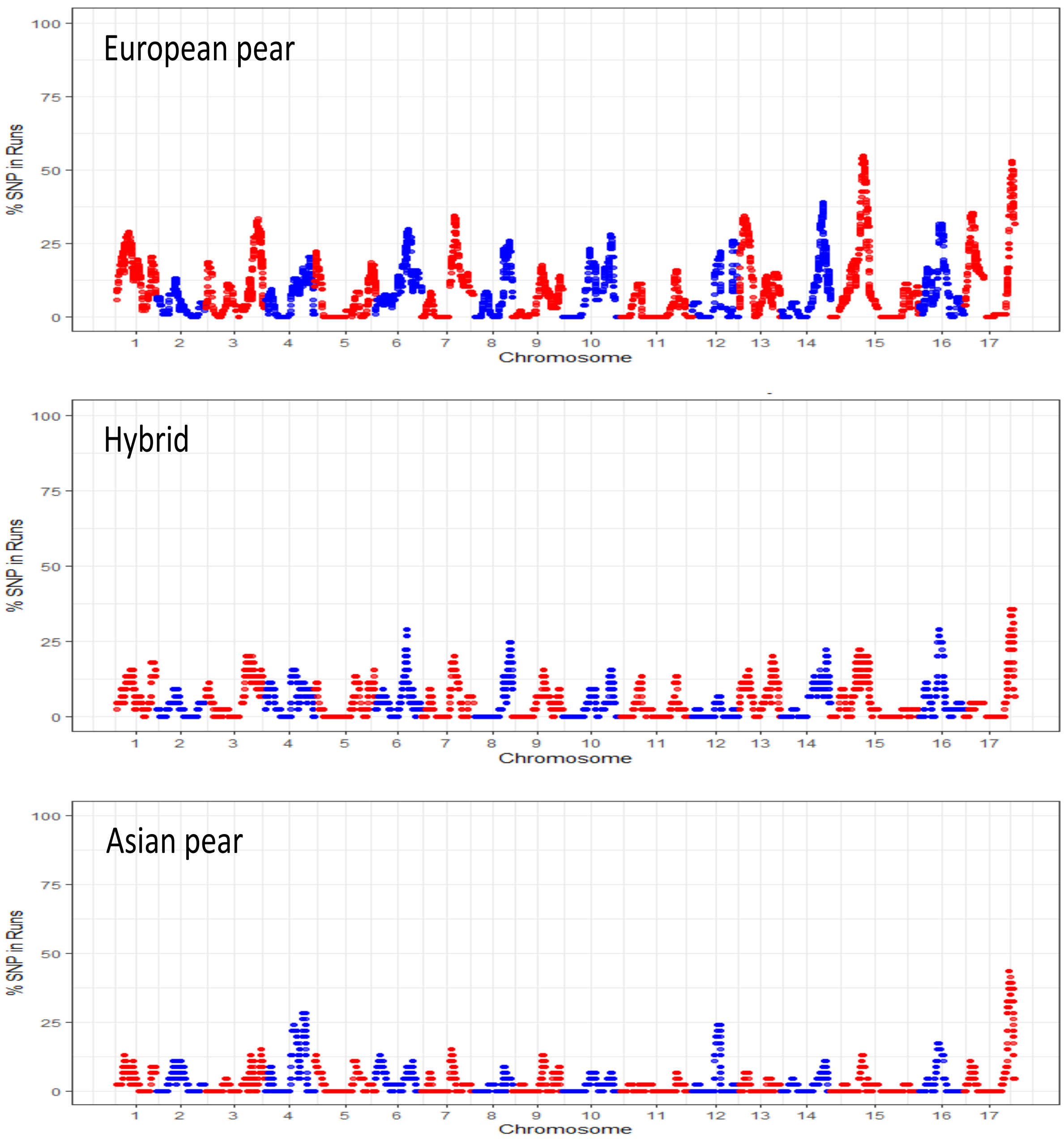

Supplementary Figure S4. The frequency (%) of single nucleotide polymorphisms (SNPs) occurrence into runs of homozygosity (ROH) islands within the Asian, European and hybrid pear (*Pyrus* spp.) population. The blue and red colour represent chromosomes.
